# Predictors of Wing Attacks by Birds Across Australian Butterflies

**DOI:** 10.1002/ece3.72596

**Published:** 2025-12-17

**Authors:** Hansani S. S. Daluwatta Galappaththige, Donald James McLean, Liisa Hämäläinen, Chathuranga Dharmarathne, Marie E. Herberstein

**Affiliations:** ^1^ School of Natural Sciences, Faculty of Science and Engineering, Macquarie University Sydney New South Wales Australia; ^2^ Department of Biological and Environmental Science University of Jyväskylä Jyväskylä Finland; ^3^ Museum of Natural History Hamburg Leibniz Institute for the Analysis of Biodiversity Change Hamburg Germany; ^4^ Department of Biology University of Hamburg Hamburg Germany

**Keywords:** avian attacks, butterfly families, predation pressure, tropics, wing damage

## Abstract

Predation pressure is a key selective force that shapes anti‐predatory traits in prey. The pressure exerted by predation can vary across biomes and different prey communities and depends on prey characteristics, such as prey size and sex. Here, the variation in predation pressure on Australian butterflies was assessed using wing damage caused by avian attacks in over 2310 individuals from five butterfly families. Temperate butterflies were less likely to receive avian attacks compared to subtropical and tropical butterflies. Male butterflies were predicted to suffer more avian attacks than females; however, our results indicated that females were more at risk. We did not find evidence of butterfly size influencing the attack likelihood. According to the regression model, Nymphalids (Browns) were more likely to receive avian attacks than Hesperiids (Skippers). The results provide interesting insights into predator–prey interactions and the factors that impact the risk of predation.

## Graphical abstract

Predation pressure can vary latitudinally and across different prey communities as well as between males and females of the same prey species. Here, the variation in predation pressure on Australian butterflies was assessed using wing damage caused by bird attacks in over 2310 individuals from five butterfly families. Temperate butterflies were less likely to receive avian attacks compared to subtropical and tropical butterflies. Females were more at risk. Nymphalids (Browns) received the most attacks. We did not find evidence of butterfly size influencing the attack likelihood.

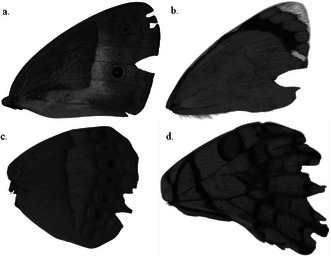

## 1 Introduction

Predators exert strong selective forces that drive the evolution of anti‐predatory traits (Lind and Cresswell 2005; Arbuckle and Speed 2015; Cooper and Blumstein 2015; Møller et al. 2017; Johnson and Belk 2020). These anti‐predatory traits reduce prey detection and predator attacks and increase prey survival (Olofsson et al. 2013). Predator community structure, predator functional and behavioural patterns, and temporal, seasonal, and spatial variation in predation pressure all significantly affect selection for anti‐predatory traits (see Kikuchi et al. 2021, for a review). Furthermore, predation pressure may vary across different prey taxa, or even between the colour morphs or sexes of the same prey species (Ohsaki 1995; Nokelainen et al. 2014), which further increases the complexity of predator–prey interactions.

Seasonally and spatially varying predation pressure can influence both overall attack rates and the types of prey being attacked (Endler and Mappes 2004; Mappes et al. 2014). Seasonal variation in predation communities, especially during their breeding cycles, may lead to changes in predator avoidance learning and the level of predation. For instance, Mappes et al. (2014) have shown increased survival of prey with conspicuous aposematic signals during non‐breeding seasons of birds, while a high detectability cost made conspicuous signals a disadvantage in seasons flushed with naïve hatchlings that have not yet learned to associate warning signals with unprofitability. In addition to seasonal variation, predator communities/assemblages and their activity and predation rates also vary spatially (Endler and Mappes 2004; Mochida 2011; Kang et al. 2017). For instance, aposematic butterflies received fewer avian attacks than non‐aposematic butterflies in geographic regions where predation pressure was lower, whereas attack rates on aposematic species increased in areas with higher predation pressure (Aluthwattha et al. 2017). Similarly, Nokelainen et al. (2014) showed that spatial heterogeneity in predator communities can maintain polymorphic warning signals in Arctiid moths. When it comes to spatial patterns of predation, global patterns are also possible. For example, lower predator abundance and density at higher latitudes (Laurila et al. 2008) are thought to result in prey experiencing lower predation pressure. There is some field‐based evidence to support this broad idea, with Roslin et al. (2017) finding an increased number of predation attacks on caterpillars at lower latitudes and lower elevations.

Predation pressure is also shaped by prey‐driven factors such as prey availability, size, type, and sex. For instance, Jara and Perotti (2010) found that smaller tadpole species were preferred over larger species by insect predators. Nokelainen et al. (2014) found that yellow colour morphs of male wood tiger moths (*Arctia plantaginis*) were attacked less by avian‐predators than white colour morphs, although this also depended on the predator community composition. Furthermore, Acharya (1995) found that male moths (Families: Noctuidae, Notodontidae, Arctiidae, and Geometridae) were eaten significantly more than female moths by insectivorous bats ( *Lasiurus cinereus* and *L. borealis* ). In addition to the prey traits, the relative abundance of prey is also important, as predators may attack more abundant prey regardless of the prey size, because more abundant prey is more economical to hunt. Thus, predation pressure is a complex phenomenon influenced by highly diverse factors that can be unravelled by broad geographic analyses.

The tropics are ideal targets when testing the impact of predation pressure as they are less seasonal than temperate regions (Adams et al. 2014). Tropics are thought to experience a high year‐round predation pressure as tropical avian‐predators are typically long‐lived and naïve juvenile predators emerge all year round. In contrast, the highly seasonal temperate regions have high proportions of naïve birds during breeding seasons, which could substantially increase predation rates (McNamara et al. 2008; Adams et al. 2014; Kikuchi et al. 2021). Understanding this temporal and spatial variation in predation pressure is critical for interpreting predation pressure across biomes. For example, Adams et al. (2014) found that tropical Ecuadorian butterflies were more colourful (more varied in hue, intensity, and saturation with a high complexity of colour patterns) than those in subtropical Florida, United States and temperate Maine, United States. While this colourfulness may reflect spatially varying selection by predation, the potential effect of sexual selection cannot be excluded, as the mechanisms were not directly tested in the study. By contrast, Australian butterflies and birds were not more colourful in terms of colour diversity, saturation, and contrast in the tropics compared to other biomes (Dalrymple et al. 2018). These contrasting patterns in Australia may be due to the specific evolutionary history of Australian butterflies and differences in their ecology and environmental pressures, such as predator communities, vegetation and sexual selection, compared with other tropical regions.

Butterflies provide an excellent model to study variation in predation pressure. They are a highly diverse taxonomic group with a range of morphological and behavioural differences among families, species, and sexes (Dapporto et al. 2019; Lindstedt et al. 2019; Medina et al. 2020), which provides an opportunity to investigate how different traits influence predation risk. Butterflies are also good models to understand how predation pressure varies temporally and spatially, as they are widely distributed across a range of climates and habitats. Furthermore, they are relatively easy to capture and predation attempts can be assessed based on wing damage, which is a commonly used indirect measure of predation in butterflies (Galappaththige 2024; Galappaththige et al. 2025). While wing damage can also result from different causes, such as ageing, collisions, and collection/handling, avian predation marks can usually be clearly identified by beak imprints, U/V‐shape cuts, symmetrical damage, triangular wing tears, and straight cuts along the major veins on the wings. In contrast, non‐predatory damage tends to be more irregular and frayed (Bowers and Wiernasz 1979; Bowers et al. 1985; Wourms and Wasserman 1985; Dennis 1992; Ota et al. 2014; Galappaththige et al. 2025). However, one limitation of this method is that wing damage measures only the individuals that survived a predation attempt in the field, since no trace remains of prey that were totally consumed; hence wing damage may underestimate true predation pressure (Galappaththige et al. 2025). Nevertheless, assessing wing damage of butterflies provides several advantages: the method can be standardised across different environments, it can be performed as a part of butterfly collection and it has no need for sophisticated instruments (Galappaththige 2024; Galappaththige et al. 2025).

Here, we analyse measures of predation pressure on butterfly communities across three Australian biomes and assess whether the risk of predation varies across taxonomic groups and sex, and butterfly size. Based on ecological theory and evidence that predator abundance and diversity are higher in the tropics (Schemske et al. 2009; Roslin et al. 2017; Nyffeler et al. 2018; Freestone et al. 2021; Erickson et al. 2025; Galappaththige et al. 2025), we predicted that tropical/subtropical butterflies would experience higher predation pressure than temperate butterflies. Here, our comparisons are based on avian attack probabilities on free‐flying butterflies and we do not directly measure predator and prey abundance in each biome.

We predicted sex‐specific variation in attack rates due to sex‐specific differences in morphology, behaviour, and reproductive function (Promislow et al. 1992; Zuk and Kolluru 1998). Male butterflies are generally more colourful than females (Kemp et al. 2005; Morehouse and Rutowski 2010) [with some exceptions such as some Satyrids (Oliver et al. 2009) and some mimetic forms of female swallowtails (Kunte 2009)], which may increase their exposure to predators. Males may also have a higher activity level than females, such as patrol flights and mate‐searching flights, which can make them more vulnerable to predation (Bergman et al. 2015; Sielezniew et al. 2020). Thus, we expected male butterflies to experience higher attack rates than females, as they are usually more active and conspicuously visible with territorial and courtship behaviours than females (Shreeve 1984; Rutowski 1997; Bergman et al. 2011; Sielezniew et al. 2020). We further predicted that larger butterflies would experience higher predation pressure than smaller butterflies due to increased detectability and greater nutritional value to predators (Chai and Srygley 1990; Halpin et al. 2013; Srygley and Kingsolver 2000).

Considering the morphological and behavioural diversity across butterfly families, we also expected to see differences in attack rates between butterfly families. For instance, Pieridae exhibit brighter and more contrasting colours with a range of hues and Papilionidae are slow‐flying butterflies that are more vulnerable to predation due to increased detectability (Chai and Srygley 1990; Srygley and Kingsolver 2000; Srygley 2004). Hesperiids are more erratic flyers with strong flight muscles (Betts and Wootton 1988; Cong et al. 2015) and Nymphalids are typically cryptic butterflies (Brakefield and Larsen 1984; Willmott 2004; Stevens 2007); hence, we expected both families to experience fewer predation attacks. The Lycaenidae family, with smaller, sometimes toxic butterflies that can have mutualistic interactions with ants (Bowers and Larin 1989; Fiedler 1991; Pierce et al. 2002; Mizokami and Yoshitama 2009; Van Der Linden et al. 2021), was expected to receive an intermediate predation risk.

## 2 Material & Methods

### 2.1 Study Sites

**FIGURE 1:**
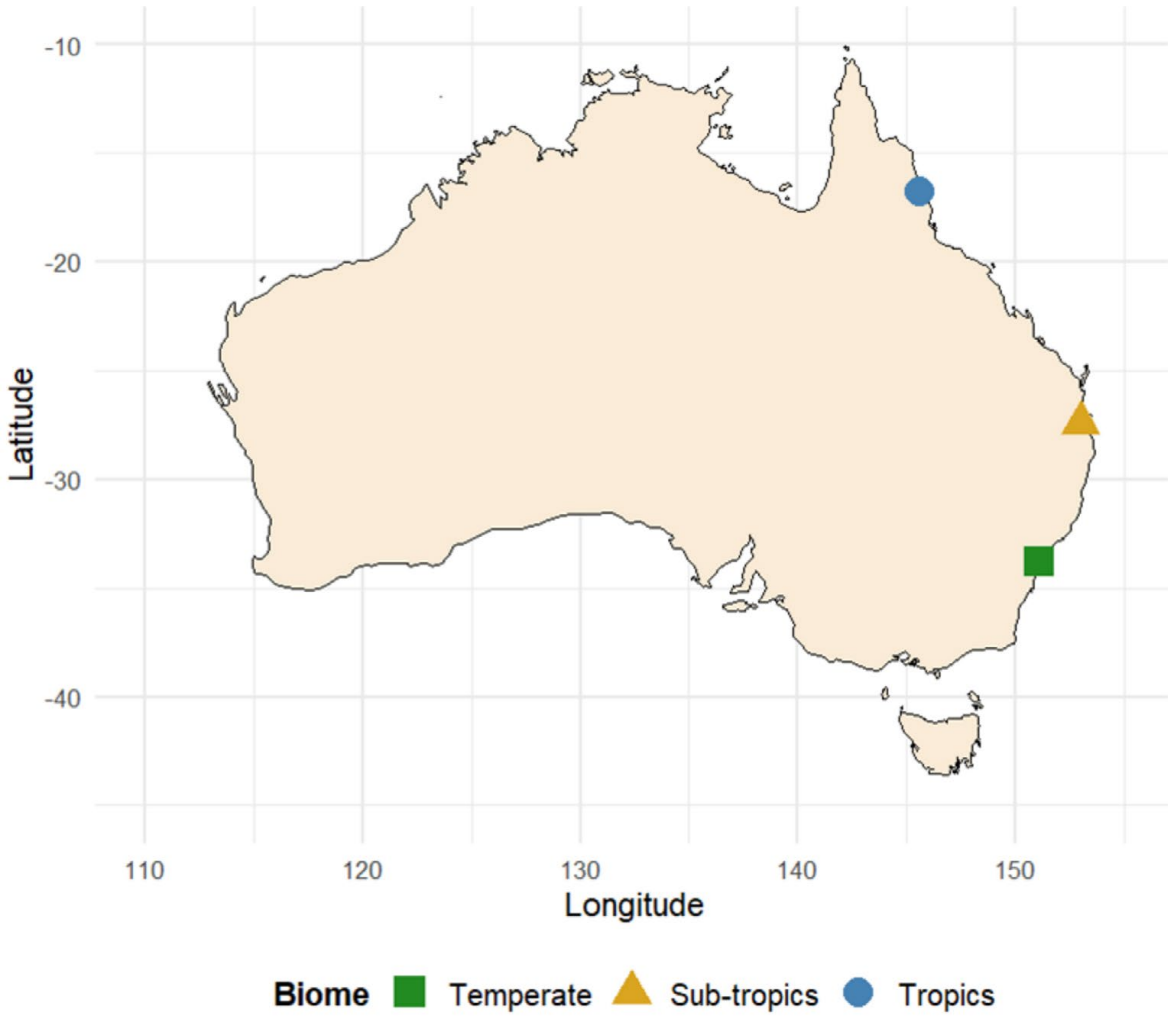
Sampling sites along the east coast of Australia spanning 1960 km of latitudinal gradient from North to South.

The study was conducted at 12 sites in Australia, representing three biomes: tropical (Cairns), subtropical (Brisbane), and temperate (Sydney) (Table 1; Figure 1). The North–South latitudinal gradient of the study sites spanned approximately 1960 km, encompassing existing Australian biogeographic classifications (temperate, subtropical, and tropical) (Thackway and Cresswell 1995; Department of Agriculture, Water and the Environment 2012) and we showed in parallel studies that the avian predator communities (i.e., insectivorous birds that potentially prey on butterflies) differed among these three biomes (higher diversity and density in tropics) (Erickson et al. 2025; Galappaththige et al. 2025). Sampling was conducted from June to November 2023 during the dry season in the tropics (Cairns: late June/early July) and spring in the subtropical and temperate biomes (Brisbane: late October/early November; Sydney: mid‐November), coinciding with peak butterfly activity in each of the biomes. Sites were selected based on safety, accessibility, and habitat suitability, representing butterfly habitats with similar vegetation in each biome, such as urban parks, forest edges and reserves.

**TABLE 1:** List of field sites where the study was conducted.

Biome	Site name	Site code	Traditional name	Coordinates
Tropics	Cairns Botanic Garden	CBG	Yidinji Land	−16.89° S, 145.74° E
Tropics	Goomboora Park	GP	Yidinji Land	−16.90° S, 145.70° E
Tropics	James Cook University Campus	JCU	Yirrganydji Land	−16.81° S, 145.68° E
Tropics	Foxton Avenue Reserve	FAR	Gungganji Land	−16.45° S, 145.37° E
Subtropics	Brisbane Botanic Gardens Mt. Coot‐tha	BBG	Jagera & Turrbal Land	−27.47° S, 152.97° E
Subtropics	Cabbage Tree Creek	CC	Jagera & Turrbal Land	−27.35° S, 153.00° E
Subtropics	Lakeside Park	LSP	Jagera & Turrbal Land	−27.22° S, 152.96° E
Subtropics	Oxley Creek	OC	Yuggera & Yugarabul Land	−27.54° S, 152.99° E
Temperate	Woo‐la‐ra Park	WLR	Cadigal Land & Cammeraygal Land	−33.83° S, 151.07° E
Temperate	Parramatta Park	PP	Dharug Land	−33.81° S, 150.99° E
Temperate	Allan Small Oval	AO	Cammeraygal Land	−33.75° S, 151.18° E
Temperate	Jubes Mountain Bike Park	JB	Dharug Land & Kuringgai Land	−33.70° S, 151.14° E

### 2.2 Butterfly Sampling and Identification

Butterflies were collected using butterfly nets (mesh size: 0.9 × 0.3 mm; hoop diameter: 456 mm) between 9.00 a.m. and 3.00 p.m. Total sampling effort at all sites was 20 h, conducted by four or five collectors. Most sites were sampled within a single day, although some sites were sampled over two days. Collected butterflies were placed inside insect envelopes (Australian Entomological Supplies Pty LTD), which were labelled with the site, collector ID, and collection date. Envelopes with butterflies were brought to the laboratory and frozen at −30°C. Butterflies were identified to species and sex level using a butterfly identification guide (Braby 2020).

### 2.3 Scoring Butterfly Wing Attacks

**FIGURE 2:**
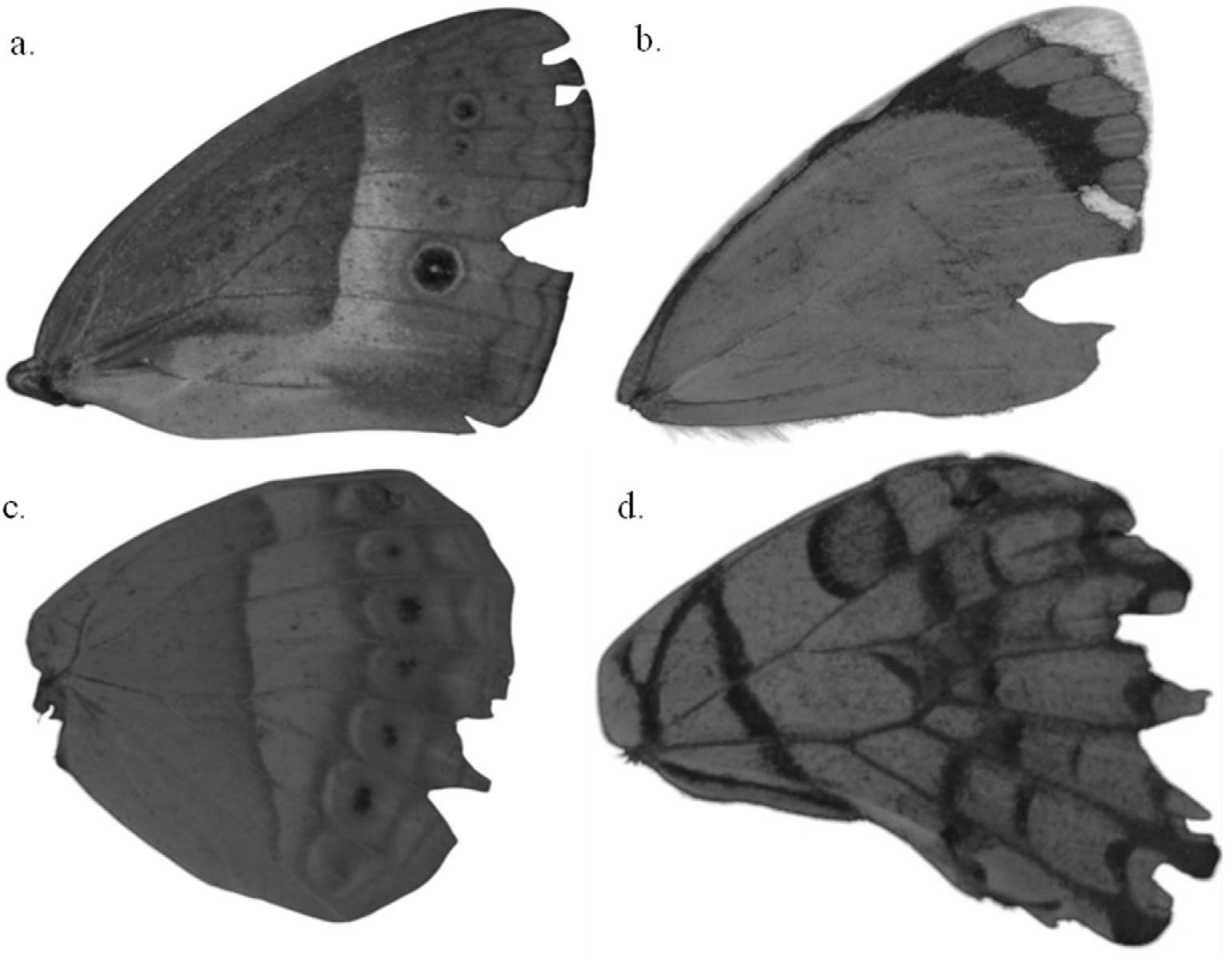
V‐shaped wing damage was assumed to be beak marks caused by avian predators (a) Forewing of *Mycalesis terminus* (b) Forewing of *Delias mysis* (c) Hindwing of *Cupha prosope* (d) Hindwing of *Papilio demoleus*.

**FIGURE 3:**
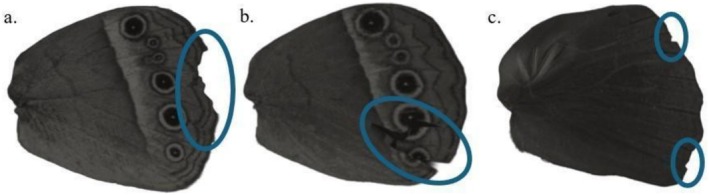
Non‐avian attacks on butterfly wings (a) Possible non‐avian predator attacks damage on the hindwing of *Mycalesis terminus* (b) Collection damage on the hindwing of *M*. *terminus*. (c) Damage possibly caused by ageing on the hindwing of *M*. *perseus*.

Butterflies were photographed with fully spread wings, allowing for assessment of both symmetrical and unilateral damage. Imaging was conducted using a Sony A7 camera (Lens: EL‐Nikkor 80 mm) and lit by three Exo Terra Intense Basking Spot Lamps (75 W). Photographs were sent to three scorers to determine whether any damage to the wings was caused by avian attacks as indicated by beak imprints, U/V‐shape cuts, triangular wing tears, straight tear cuts along the major veins and on the wings (Bowers and Wiernasz 1979; Bowers et al. 1985; Wourms and Wasserman 1985; Dennis 1992; Ota et al. 2014; Figure 2) or due to non‐avian predator attacks, collection damage, or other causes (e.g., ageing, mechanical damage) (Figure 3). The butterfly was considered to be attacked if it was scored to have an avian attack mark in any of the fore or hindwings. Scorers were trained using published articles to ensure that scoring was consistent (Bowers and Wiernasz 1979; Miyata 2005; Ota et al. 2014; Galicia et al. 2019) and they were unaware of the site from which the butterflies were collected. Wing attack score was assigned based on the majority opinion (⅔), which helped to reduce the individual bias in scoring (Galappaththige et al. 2025). Seventy‐seven per cent of avian attacks were agreed on by all three scorers, while the remaining 23% of avian attacks were agreed on by two scorers. Finally, the wing attack rate per biome was calculated.

### 2.4 Data Analysis

Data analyses were performed in R (Version 4.2.2; R Core Team 2020). A generalised linear mixed model (GLMM), fitted using the lme4 package (Bates et al. 2015) with a binomial error distribution and logit link function, was used to assess the differential predation pressure on butterflies across the three biomes. Individual butterflies were used as the unit of analysis of predation. Butterfly species represented by a single individual across all biomes were excluded from the statistical analyses. The response variable was a two‐column matrix combining counts of avian‐attacked and non‐attacked butterflies at each site, constructed using the cbind() function. *Biome* (tropics, subtropics, and temperate), *butterfly family* (Hesperiidae, Lycaeniade, Nymphalidae, Papilionidae, and Pieridae), *sex,* and *wingspan* (as a proxy for body size) were included as predictor variables. Wingspan data for each species and sex were obtained from ‘The Complete Field Guide to Butterflies of Australia’ (Braby 2020). *Site* and *butterfly species* were included as random variables (GLMM1; Table 3).

Several logistic regression models were also run without *site* (GLMM2; Table S1), without *butterfly species* (GLMM3; Table S2), and without *butterfly family* (GLMM4; Table S3). The effect of family–sex interactions (GLMM5; Table S4) and family–biome interactions (GLMM6; Table S5) on wing attacks was also tested.

Post hoc comparisons were performed using the *emmeans* package (Lenth 2024) based on estimated marginal means to estimate the differences between categorical variables (i.e., *Biome*, *butterfly family,* and *sex*). Here, Tukey tests were used to reduce type I error when comparing more than two levels, such as the biome and butterfly family.

Finally, the relationship between the relative abundance and relative avian attack rates of butterfly families in each biome was tested using Pearson correlation analyses.

## 3 Results

### 3.1 Butterfly Community Composition

**FIGURE 4:**
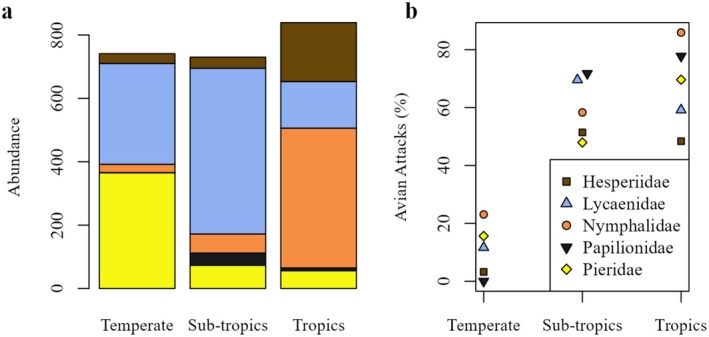
(a) Number of individuals captured in the five butterfly families across Australian biomes (b) Wing attacks of butterfly families across Australian biomes.

A total of 2310 butterflies representing 95 species from five families: Hesperiidae, Lycaenidae, Nymphalidae, Papilionidae, and Pieridae, were collected across three biomes in Australia during our study (Table S11; Figure 4a). The majority of the captured individuals were males (Male: ~62%; Female: ~38%). The highest number of butterflies was captured in the tropics, followed by the subtropics, and temperate biomes. Males were captured more often than females in all three biomes (Table 2).

**TABLE 2:** Composition of butterflies collected from three Australian biomes showing the number of males and females collected from tropical, subtropical, and temperate sites. The percentages in brackets indicate the proportion of each sex relative to the total collection of butterflies in that respective biome. The percentage of the total collection indicates the percentage of collected butterflies across all three biomes.

Biome	Male	Female	Total	% of the total collection
Tropics	526 (63%)	313 (37%)	839	36
Subtropics	425 (58%)	305 (42%)	730	32
Temperate	489 (66%)	252 (34%)	741	32

All five butterfly families listed above were found in all three biomes with variable abundances (Figure 4a). In the tropics, Nymphalidae was the dominant family (~53% of the collected butterflies) while Papilionidae was the least abundant (~1%). In the subtropics, Lycaenidae was the most abundant family (~72% of the collected butterflies), whereas Hesperiidae was the least (~5%). Pieridae was the most abundant (~49% of the collected butterflies) in the temperate biome, whereas Papilionidae was the least abundant (~0.13%).

The highest number of butterfly species was recorded in the tropics, with 54 species, where *Mycalesis terminus* (Orange Bush brown) was the dominant species (~23% of the collected butterflies). The tropics also contained 17 species that were only represented by a single individual (Table S11). In the subtropics, 37 butterfly species were collected, with *Zizula hyrax* (Dainty Grass‐blue) being the most abundant (~35% of the collected butterflies) and 11 species only being represented by a single individual. The lowest number of butterfly species was collected in the temperate biome, with 27 species. *Pieris rapae* (Cabbage White) was the dominant species (~49% of the collected butterflies) and 12 species were represented by a single specimen (Table S11).

### 3.2 Avian Predation on Butterflies

A total of 1183 butterflies (~51% of the total butterfly collection) were scored as having avian attack marks on their wings (Forewings: 41%; Hindwings: 59%; Figure 4b). Females received significantly higher avian attacks than males (Tables 4 and 5). Across all collected species, *Zizula hylax* had the highest rate of attack marks (~62% of individuals attacked), with most of the attacks on females (~71%). All butterfly families received avian attacks in a variable proportion in each biome (Figure 4b).

Butterflies from the temperate biome had the lowest avian attacks (with only ~13% of all individuals carrying bird marks on their wings; Tables 3 and 4; Table S12). Both sexes were attacked at similar rates (~14%). Here, *Junonia villida* (Meadow Argus) was recorded as the highest avian‐attacked species (Table 5), with female *Junonia villida* more likely to be attacked (~67%) than males (~33%).

**TABLE 3:** GLMM1 showing avian attack probabilities on Australian butterflies in 2023, explained by *biome*, *butterfly family*, *sex,* and *wingspan* (random effects: *Sites*; standard deviation: 0.518; *butterfly species*; standard deviation: 0.586; AIC: 691.84). Intercept gives the estimate (logit) for the attack probabilities on females of the family Hesperiidae in the subtropical region.

Source	Estimate	SE	*Z*	*p*
Intercept	0.888	0.512	1.737	0.082
Family Lycaenidae	0.955	0.373	2.561	0.010*
Family Nymphalidae	1.832	0.409	4.475	< 0.001***
Family Papilionidae	1.704	0.748	2.278	0.023*
Family Pieridae	0.801	0.451	1.776	0.076
Sex Male	−0.484	0.114	−4.250	< 0.001***
Biome Temperate	−2.639	0.411	−6.427	< 0.001***
Biome Tropics	0.118	0.410	0.287	0.774
Wingspan	−0.020	0.011	−1.833	0.067

*Note:* * and *** denote significance.

**TABLE 4:** Pairwise post hoc comparisons for biomes, butterfly families, and sex showing odds ratio generated from *emmeans*. The odds ratio shows the relative likelihood of avian attacks between two categories.

Contrast	Odds ratio	SE	*Z*	*p*
Subtropics vs. Temperate	13.998	5.748	6.427	< 0.001***
Subtropics vs. Tropics	0.889	0.364	−0.287	0.956
Temperate vs. Tropics	0.064	0.027	−6.458	< 0.001***
Hesperiidae vs. Lycaenidae	0.385	0.144	−2.561	0.078
Hesperiidae vs. Nymphalidae	0.160	0.066	−4.475	< 0.001***
Hesperiidae vs. Papilionidae	0.182	0.136	−2.278	0.152
Hesperiidae vs. Pieridae	0.449	0.202	−1.776	0.388
Lycaenidae vs. Nymphalidae	0.416	0.200	−1.822	0.361
Lycaenidae vs. Papilionidae	0.473	0.382	−0.927	0.886
Lycaenidae vs. Pieridae	1.166	0.577	0.310	0.998
Nymphalidae vs. Papilionidae	1.136	0.668	0.217	1.000
Nymphalidae vs. Pieridae	2.803	1.128	2.560	0.078
Papilionidae vs. Pieridae	2.467	1.664	1.339	0.667
Female vs. Male	1.620	0.184	4.250	< 0.001***

*Note:* *** denotes significance.

**TABLE 5:** Species with avian attacks in three Australian biomes. Only species with more than six collected specimens are listed here. ‘Attacked individuals’ indicate the number of individuals with avian attack marks, with the total number of individuals collected from each species in brackets. Butterfly families: H: Hesperiidae; L: Lycaenidae; N: Nymphalidae; Pa: Papilionidae; Pi: Pieridae.

Biome	Species	Family	Attacked individuals	% of attacked individuals	Wingspan (mm)	
					Male	Female
Tropics	*Mycalesis terminus*	N	167 (192)	87%	38	43
	*Junonia hedonia*	N	58 (63)	92%	50	51
	*Mycalesis perseus*	N	58 (75)	77%	35	38
	*Zizina otis*	L	41 (82)	50%	20	23
	*Eurema hecabe*	Pi	29 (38)	76%	37	40
	*Mycalesis sirius*	N	24 (31)	77%	38	42
	*Telicota mesoptis*	H	25 (63)	40%	26	26
	*Zizula hylax*	L	32 (48)	67%	15	16
	*Cupha prosope*	N	21 (21)	100%	47	53
	*Suniana sunias*	H	24 (47)	51%	21	22
	*Melanitis leda*	N	12 (12)	100%	60	63
	*Arrhenes dschilus*	H	10 (14)	71%	26	29
	*Ypthima arctous*	N	14 (17)	82%	28	32
	*Sabera dobboe*	H	8 (9)	89%	29	32
	*Delias mysis*	Pi	9 (12)	75%	57	56
	*Pelopidas agna*	H	7 (17)	41%	33	36
	*Telicota ancilla*	H	6 (13)	46%	29	31
Subtropics	*Zizula hylax*	L	156 (255)	61%	15	16
	*Zizina otis*	L	97 (128)	76%	20	23
	*Leptotes plinius*	L	59 (78)	76%		
	*Theclinesthes onycha*	L	35 (39)	90%	22	23
	*Papilio demoleus*	Pa	22 (28)	79%	72	75
	*Pieris rapae*	Pi	24 (50)	48%	44	44
	*Nacaduba berenice*	L	7 (11)	64%	22	22
	*Toxidia peron*	H	5 (8)	63%	29	30
Temperate	*Pieris rapae*	Pi	57 (364)	16%	44	44
	*Zizina otis*	L	33 (290)	11%	20	23
	*Junonia villida*	N	3 (10)	30%	40	43
	*Candalides hyacinthinus*	L	2 (16)	13%	28	28

About 68% (Table S12) of butterflies from the subtropics had avian attack marks; again, females suffered higher attack rates (~77%, Tables 3 and 4). *Theclinesthes onycha* (Cycad Blue) was the most attacked butterfly species (~90%; Table 5); 100% of male *T*. *onycha* had been attacked by avian predators.

Butterflies from the tropics had the highest rate of avian attacks (~73%; Table S12), with females suffering the highest attack rate (~74%). The tropical species with the highest attack rates were *Melanitis leda* (Evening Brown) and *Cupha prosope* (Bordered Rustic) (100% of collected individuals of these species bore wing attack damage; Table 5).

The logistic regression model (GLMM1) with *site* and *butterfly species* as random variables and the *butterfly family* as a predictor variable (along with *biome*s, *sex,* and *wingspan* as predictor variables) was a better fit (Table 3) than the model without *site* (GLMM2; Tables S1 and S6), the model without *butterfly species* (GLMM3; Tables S2 and S7), or the model without *butterfly family* (GLMM4; Tables S3 and S8). Inclusion of butterfly family*sex did not improve the model fit (GLMM5; Tables S4 and S9) (Akaike 1974). Furthermore, the model with butterfly family*biome (GLMM6; Tables S5 and S10) was statistically significant compared to GLMM1 (Table 3). However, GLMM6 generated a model with a very high standard error for some non‐significant interaction terms, likely due to overfitting or sparse data for some butterfly families. Considering the complexity of GLMM6, we used GLMM1 as the final model, as it did not show overdispersion (Pearson residuals; dispersion ratio = 1.00, *p* = 0.48).

**FIGURE 5:**
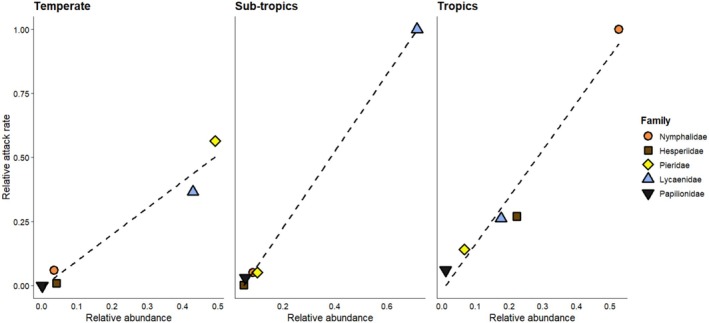
Relationship between relative abundance and relative avian attack rates at a butterfly family level in (a) temperate biome, (b) subtropical biome, and (c) tropical biome.

Tropical and subtropical butterflies experienced similar probabilities of avian attacks (Tables 3 and 4) but received significantly higher avian attacks compared to the temperate butterflies (Table 4). Females experienced significantly higher avian attacks than males (Tables 3 and 4). The probability of avian attacks differed among butterfly families (Tables 3 and 4; Table S12). Hesperiidae received significantly fewer avian attacks compared to Nymphalidae (Table 4), which showed the highest likelihood of receiving avian attacks (Table 3). Papilionidae showed a statistically significant difference in avian attacks from Hesperiidae in the regression model (Table 3). However, both families showed a similar attack probability in pairwise comparisons (Table 4), possibly due to the lack of statistical control for other effects, such as biome, sex, and family, which were included in the regression model. Lycaenidae, Pieridae, and Papilionidae were similar in attack probabilities in pairwise comparisons (Table 4), although they showed some significant differences when controlling for biome, sex, and other factors (Table 3). Wingspan was not significantly related to the attack rate (Table 3). We found strong correlations between relative butterfly abundance and relative avian attack rates of butterfly families in each biome [Pearson correlation coefficients: temperate: 0.979 (*p* value: 0.004, df: 3); subtropics: 0.999 (*p* value: < 0.001, df: 3); tropics: 0.979 (*p* value: 0.004, df: 3); Figure 5].

## 4 Discussion

Our study aimed to assess the impact of biome, taxonomy, size, and sex‐specific variation in avian predation on Australian butterflies. Our results support the idea that predator attacks are generally lower in temperate biomes. Of the five butterfly families surveyed, Nymphalidae were the most attacked. Contrary to our predictions, we did not find that larger butterflies suffered more wing attacks nor were male butterflies more likely to be attacked than females. This suggests that predation pressure on butterflies varies across biomes and between the sexes, and taxonomic groups, indicating different risks of predation on butterfly communities.

The overall wing attack rate in our study was markedly higher than previously reported, with more than half of our collected butterflies having been attacked by birds (~51% pooling all biomes). In a similar study in Japan, the wing attack rate by birds and mantises was comparatively low, with only ~7% of individuals attacked (Ota et al. 2014). Differences in butterfly‐prey and avian‐predator diversity, density, and composition in different geographic regions and seasons are likely to underlie the observed variation in wing attacks, both across different studies and within our study. In our study, seasonal variation was minimal among biomes, as the sampling was conducted during the peak activity of butterflies in each biome. It is also possible that our assumed avian attack rates were overestimated however, since we had multiple scorers, we reduced the likelihood of systematic bias; and more than 75% of the avian wing attacks were agreed upon by all three scorers. Furthermore, even if we overestimated the attack rates, any such bias would likely affect all groups similarly, meaning that the comparative patterns between biomes, sex, and family would still indicate biological differences. Additionally, butterflies attacked by predators might be more easily captured due to impaired flight performance, but this would not explain the lower attack rate in previous studies that used similar capture methods. In addition, we captured active flying butterflies and found wing damage mainly on hind wings, which is likely to be less detrimental to flight ability than damage to the forewings (Jantzen and Eisner 2008).

We predicted higher wing attacks in the tropics and subtropics compared to temperate regions, as predator abundance and diversity at lower latitudes are thought to be greater, resulting in higher predation pressure according to ecological theory and field evidence (Schemske et al. 2009; Nyffeler et al. 2018; Zvereva et al. 2019; Erickson et al. 2025; Galappaththige et al. 2025). This prediction was supported, with temperate butterflies experiencing fewer wing attacks than subtropical and tropical butterflies. Similar to our study, Roslin et al. (2017) found that predation on caterpillars by arthropod predators increased towards the equator (conducted across six continents: North America, South America, Europe, Africa, Asia, and Australia). However, they did not find a latitudinal trend of predation by avian and mammalian predators. Zvereva et al. (2019) found decreased arthropod predation attacks on artificial caterpillars towards the higher latitudes, but a contradictory increase in avian predation on artificial caterpillars towards the higher latitudes (conducted across four continents: South America, Europe, Africa, and Asia). Most recently, Stefanescu et al. (2025) found an increased pattern of predation risk (wing attacks) in a migratory butterfly, *Vanessa cardui* , towards the lower latitudes (conducted in the northern Mediterranean region, Maghreb, and Sub‐Saharan West Africa), similar to our study. Overall, previous studies, along with our results, suggest that there is a variation in predation pressure on butterflies across biomes and that temperate regions are likely to experience comparatively lower predation pressure. This geographical variation of predation pressure may have implications for the understanding of the biogeography of predator–prey dynamics and the evolution of anti‐predatory traits on a broader scale.

Differences in predation pressure among the collected butterfly families were also predicted due to the diversity in butterfly morphology. We found differences in butterfly abundance and species composition between the three biomes with varied levels of wing attacks. Overall, the Family Nymphalidae had the highest number of wing attacks according to the regression model, although pairwise comparison of families found the differences to be non‐significant, except that the attack rate of Nymphalids was significantly higher than for Hesperiids. Similar to our results, Ota et al. (2014) found differential wing attacks on butterfly families in Japan, where Papilionidae and Nymphalidae were the most attacked. Experimental tests have further shown that butterflies with longer forewings experienced more attacks (Kiritani et al. 2013) and the increased avian attacks on Nymphalids may be due to a preference by predators towards larger prey (Ohsaki 2005). In contrast, our study did not find any significant relationship between wingspan and wing attack rates. This implies that although some larger butterfly taxa are likely preferred by avian predators, wingspan alone may not play a significant role without considering overall morphology and behaviour. Additionally, our analyses identified a strong positive correlation between relative avian attack rates and the relative abundance of each family in each biome, evidencing that predation attacks are dependent on the availability of the butterfly prey. This may partly explain the variation of wing attacks among butterfly families, with Nymphalids having the highest abundance and avian attack rates, especially in the tropics.

Skippers (Hesperiids) received the lowest number of attacks according to the regression model (although pairwise comparison only found their attack rate to be significantly lower than that of Nymphalidae), which may be due to their often cryptically coloured (brown) phenotypes and strong flight ability (Betts and Wootton 1988; Cong et al. 2015). Lycaenids were less frequently attacked than Hesperiids according to the regression model, which may reflect their known toxicity (Bowers and Larin 1989; Mizokami and Yoshitama 2009; Van Der Linden et al. 2021), although pairwise comparisons did not find significant differences with other families. However, *T*. *onycha* (Cycad Blue), which feeds on toxic cycad plants (Whitaker and Salzman 2020), surprisingly received the highest rate of avian attacks in the entire subtropical butterfly community. These toxic cycad blues may have been taste‐rejected by avian predators once they detected the unpalatable toxin (Skelhorn and Rowe 2006). Papilionids had the second highest predicted avian attack rates according to the regression model (although pairwise comparisons did not find significant differences with other families), which may reflect their unpalatability to avian predators or lower abundance. Pierids are often very colourful (e.g., *Eurema hecabe*, *Delias mysis,* and *Belenois java*) and potentially aposematic, deterring predator attacks (Lyytinen et al. 1999; Wee and Monteiro 2017). However, while the regression model showed some differences, pierids received similar attack rates as other families, in opposition to our prediction. While we collected several species of colourful pierids, that is, *Eurema* spp., *Elodina* spp., *Cepora* sp., *Catopsilia* sp., *Belenois* sp., *Delias* spp. and *Pieris* sp., the sample size was too low for further analysis. Overall, differential avian attack rates in different butterfly families suggest that their relative abundance, size, and anti‐predatory traits, such as toxicity and conspicuous/cryptic colouration, likely affect the willingness or ability of avian predators to attack and the likelihood of the butterfly surviving the attack.

The variation in attack rates between butterfly sexes was also tested in our study. However, contrary to our prediction, males were less likely to receive avian attacks compared to females. Our results align with Ohsaki (1995) and Kiritani et al. (2013), who also found female‐biased predation attacks on butterflies. Females may be more vulnerable to attack because they are relatively slow fliers carrying heavy eggs and often constitute a more nutritious meal for predators (Ohsaki 1995; Almbro and Kullberg 2012; Maisonneuve et al. 2022). Furthermore, variability of colouration between sexes may affect the risk of predation due to detectability to predators (Kunte 2008; Morehouse and Rutowski 2010; Finkbeiner et al. 2014). However, in our case, many species were not sexually dimorphic, which reduces the effect on sex‐specific predation based on visual cues for predators. It is more likely that sex‐specific behaviour results in different risks of attack (Galicia et al. 2019; Molleman et al. 2020). We did not find differences in sex‐specific attack rates across different butterfly families, implying that avian predation on males and females is consistent regardless of the taxonomic level.

While avian predation on caterpillars is widely documented (Hooks et al. 2003; Lichter‐Marck et al. 2015; Zvereva et al. 2019), there is a lack of studies on avian predation on adult butterflies, especially in Australia. We still know very little about which Australian bird species directly feed on butterflies. Nevertheless, in our parallel studies, we recorded potential predators of butterflies such as fantails (*Rhipidura* spp.) and thornbills (*Acanthiza* spp.) (Erickson et al. 2025; Galappaththige et al. 2025), but we did not directly observe them consuming real butterflies, as predation events in the wild are difficult to observe. It is therefore unclear whether different bird species are targeting different sexes or families of butterflies. Additionally, direct observations of attacks can establish if butterflies are more vulnerable during flight or while perching. However, in our study, we noted that hind wings were more likely to be damaged by bird predators, implying that more individuals were attacked during flight.

Overall, our results support the prediction that there is variation in predation pressure on butterflies across Australian biomes, with temperate regions experiencing significantly lower attacks than the tropics/subtropics. Our results also revealed sex‐specific and family‐specific variation in predation on butterflies. However, we did not find evidence for size‐specific variation in predation pressure. Furthermore, we recognise a key limitation of our methodology in that attack marks only provide evidence of prey surviving an encounter with predators. This can underestimate the predation pressure on species/families/sexes that are consumed and can incorrectly imply low predation rates for some taxa due to a lack of predation evidence. These limitations may be overcome by integrating several complementary approaches, such as assessing predation attacks on artificial replicas, predator surveys, or camera trapping (Galappaththige 2024; Galappaththige et al. 2025). However, despite these methodological limitations, avian attack rates in our study are still relevant and reflect biologically meaningful evidence and insights into varied predator–prey interactions across biomes.

Our study provides the basis for future studies to experimentally test predator choices more directly. Furthermore, the prey traits of different families and sexes (e.g., colour patterns, anti‐predatory traits) can drive the variation of predation pressure, which could be experimentally tested in future investigations. Finally, field‐based predation studies can be integrated with laboratory experiments, such as predator diet analyses, to obtain a deeper understanding of predator–prey interactions. Overall, our work highlights the importance of prey and climate variation in predicting and describing predator–prey interactions and provides the basis for future studies to experimentally test predator choices more directly.

## Author Contributions

**Hansani S. S. Daluwatta Galappaththige:** conceptualization (lead), data curation (lead), formal analysis (lead), funding acquisition (lead), investigation (lead), methodology (lead), project administration (lead), resources (lead), validation (lead), visualization (lead), writing – original draft (lead), writing – review and editing (lead). **Donald James McLean:** data curation (supporting), formal analysis (supporting), resources (supporting), validation (supporting), visualization (supporting), writing – review and editing (supporting). **Liisa Hämäläinen:** formal analysis (supporting), methodology (supporting), writing – review and editing (supporting). **Chathuranga Dharmarathne:** methodology (supporting), writing – review and editing (supporting). **Marie E. Herberstein:** conceptualization (lead), data curation (supporting), formal analysis (lead), funding acquisition (lead), investigation (supporting), methodology (lead), project administration (lead), supervision (lead), validation (supporting), visualization (supporting), writing – review and editing (supporting).

## Funding

The project was funded by the Australian Research Council grant (#DP22010232) and the International Macquarie Research Excellence Scholarship (‘iMQRES’: #20235660). LH is currently funded by the Academy of Finland Research Fellowship (#355869) and a Horizon Europe MSCA Postdoctoral Fellowship (#101151042).

## Disclosure

Diversity Statement: We respect and support diversity, equity and inclusion in Science (Rößler et al. 2020). The authors originate from different countries (Sri Lanka, Austria, Australia, and Finland), representing different cultures, ethnicities, backgrounds, genders, and career stages (Ph.D. candidates, postdoctoral fellows, early career researchers, and professors). At least one author identifies as a sexual minority.

## Conflicts of Interest

The authors declare no conflicts of interest.

## Supporting information


**Appendix S1:** ece372596‐sup‐0001‐AppendixS1.docx.

## Data Availability

The data underlying the work are available in the main article and Supporting Information.
