# Supplementary material for: Predictors of Wing Attacks by Birds Across Australian Butterflies
Source: Ecol Evol. 2025 Dec 17;15(12):e72596. doi: 10.1002/ece3.72596 (PMC12711438; doi:10.1002/ece3.72596)
Supplement: Supplementary file 1 — Appendix S1: ece372596‐sup‐0001‐AppendixS1.docx. [file ECE3-15-e72596-s001.docx]

**Table S1** GLMM2 testing the effect of *biomes*, *butterfly family*, *sex*, and *wingspa*n on the rate of avian attacks on Australian butterflies in 2023 without ‘*site*’ as a random variable (AIC: 720.26) (Random variable: *butterfly species*: standard deviation: 0.473)

| **Source** | **Estimate** | **SE** | **Z** | **P** |
| --- | --- | --- | --- | --- |
| Intercept | 0.643 | 0.392 | 1.638 | 0.101 |
| Family Lycaenidae | 0.886 | 0.328 | 2.702 | **0.007 **** |
| Family Nymphalidae | 1.776 | 0.361 | 4.916 | **< 0.001 ***** |
| Family Papilionidae | 1.590 | 0.667 | 2.384 | **0.017 *** |
| Family Pieridae | 0.758 | 0.401 | 1.890 | 0.059 |
| Sex Male | -0.483 | 0.110 | -4.378 | **< 0.001 ***** |
| Biome Temperate | -2.495 | 0.173 | -14.386 | **< 0.001 ***** |
| Biome Tropics | 0.051 | 0.164 | 0.311 | 0.756 |
| Wingspan | -0.014 | 0.009 | -1.513 | 0.130 |

**Table S2** GLMM3 testing the effect of *biomes*, *butterfly family*, *sex*, and *wingspan* on the rate of avian attacks on Australian butterflies in 2023 without ‘*butterfly species*’ as a random variable (AIC: 711.86). (Random effect by *site*: standard deviation: 0.413)

| **Source** | **Estimate** | **SE** | **Z** | **P** |
| --- | --- | --- | --- | --- |
| Intercept | 0.739 | 0.359 | 2.060 | **0.039 *** |
| Family Lycaenidae | 0.694 | 0.193 | 3.596 | **< 0.001 ***** |
| Family Nymphalidae | 2.081 | 0.244 | 8.537 | **< 0.001 ***** |
| Family Papilionidae | 1.813 | 0.510 | 3.552 | **< 0.001 ***** |
| Family Pieridae | 1.270 | 0.243 | 5.231 | **< 0.001 ***** |
| Sex Male | -0.467 | 0.108 | -4.309 | **< 0.001 ***** |
| Biome Temperate | -2.635 | 0.333 | -7.910 | **< 0.001 ***** |
| Biome Tropics | 0.119 | 0.334 | 0.356 | 0.722 |
| Wingspan | -0.020 | 0.007 | -2.884 | **0.004**** |

**Table S3** GLMM4 testing the effect of *biomes*, *sex*, and *wingspan* on the rate of avian attacks on Australian butterflies in 2023 without ‘*butterfly family*’ as a response variable (AIC: 701.79). (Random effect by *site*: standard deviation: 0.544; *butterfly species*: standard deviation: 0.901)

| **Source** | **Estimate** | **SE** | **Z** | **P** |
| --- | --- | --- | --- | --- |
| Intercept | 1.066 | 0.477 | 2.236 | **0.025 *** |
| Sex Male | -0.451 | 0.115 | -3.936 | **< 0.001 ***** |
| Biome Temperate | -2.643 | 0.430 | -6.150 | **< 0.001 ***** |
| Biome Tropics | 0.050 | 0.427 | 0.116 | 0.908 |
| Wingspan | 0.002 | 0.008 | 0.200 | 0.842 |

**Table S4 GLMM5** showing the effects of interaction between *butterfly family* and *sex* on avian attacks on Australian butterflies in 2023 (AIC: 691.93). (Random effect by *site*: standard deviation: 0.509; *butterfly species*: standard deviation: 0.574)

| **Source** | **Estimate** | **SE** | **Z** | **P** |
| --- | --- | --- | --- | --- |
| Intercept | 0.845 | 0.527 | 1.603 | 0.109 |
| Family Lycaenidae | 1.139 | 0.417 | 2.734 | **0.006 **** |
| Family Nymphalidae | 1.942 | 0.469 | 4.141 | **< 0.001 ***** |
| Family Papilionidae | 2.270 | 0.899 | 2.525 | **0.012 *** |
| Family Pieridae | 0.404 | 0.517 | 0.782 | 0.434 |
| Sex Male | -0.379 | 0.295 | -1.286 | 0.198 |
| Biome Temperate | -2.637 | 0.405 | -6.508 | **< 0.001 ***** |
| Biome Tropics | 0.132 | 0.404 | 0.327 | 0.744 |
| Wingspan | -0.021 | 0.011 | -1.968 | **0.049 *** |
| FamilyLycaenidae: SexMale | -0.314 | 0.336 | -0.936 | 0.349 |
| FamilyNymphalidae: SexMale | -0.135 | 0.402 | -0.335 | 0.738 |
| FamilyPapilionidae: SexMale | -0.845 | 0.865 | -0.976 | 0.329 |
| FamilyPieridae:SexMale | 0.544 | 0.412 | 1.322 | 0.186 |

**Table S5 GLMM6** showing the effects of interaction between *butterfly family* and *biome* on avian attacks on Australian butterflies in 2023 (AIC: 675.90). (Random effect by *site*: standard deviation: 0.574; butterfly species: standard deviation: 0.447)

| **Source** | **Estimate** | **SE** | **Z** | **P** |
| --- | --- | --- | --- | --- |
| Intercept | 0.609 | 0.599 | 1.018 | 0.309 |
| Family Lycaenidae | 0.965 | 0.477 | 2.022 | **0.043 *** |
| Family Nymphalidae | 0.294 | 0.722 | 0.407 | 0.684 |
| Family Papilionidae | 0.615 | 0.858 | 0.716 | 0.474 |
| Family Pieridae | -0.124 | 0.602 | -0.206 | 0.837 |
| Biome Temperate | -19.050 | 2386.000 | -0.008 | 0.994 |
| Biome Tropics | -0.094 | 0.590 | -0.159 | 0.874 |
| Sex Male | -0.441 | 0.114 | -3.868 | **< 0.001** *** |
| Wingspan | -0.003 | 0.113 | -0.304 | 0.761 |
| FamilyLycaenidae: BiomeTemperate | 15.640 | 2386.000 | 0.007 | 0.995 |
| FamilyNymphalidae: BiomeTemperate | 17.260 | 2386.000 | 0.007 | 0.994 |
| FamilyPapilionidae: BiomeTemperate | 0.774 | 10940.000 | 0.000 | 1.000 |
| FamilyPieridae: BiomeTemperate | 17.700 | 2386.000 | 0.007 | 0.994 |
| FamilyLycaenidae: BiomeTropics | -0.278 | 0.476 | -0.584 | 0.559 |
| FamilyNymphalidae: BiomeTropics | 1.566 | 0.661 | 2.369 | **0.018*** |
| FamilyPapilionidae: BiomeTropics | 0.462 | 1.078 | 0.428 | 0.669 |
| FamilyPieridae: BiomeTropics | 0.763 | 0.694 | 1.100 | 0.271 |

**Table S6** Comparison of GLMM1 (includes response variables: *biomes*, *butterfly family*, *sex*, and *wingspan*; random variables: *sites*, and *butterfly species*) and GLMM2 (includes response variables: *biome*, *butterfly family*, *sex*, and *wingspan*; random variable: *butterfly species*)

| **Source** | **AIC** | **BIC** | **Loglik** | **Deviance** | **Chi-squared (χ²)** | **Df** | **P** |
| --- | --- | --- | --- | --- | --- | --- | --- |
| GLMM1 | 691.84 | 730.80 | -334.92 | 669.84 | 30.417 | 1 | **< 0.001** *** |
| GLMM2 | 720.26 | 755.67 | -350.13 | 700.26 | - | - | - |

**Table S7** Comparison of GLMM1 (includes response variables: *biomes*, *butterfly familiy*, *sex*, and *wingspan*; random variables: *sites*, and *butterfly species*) and GLMM3 (includes response variables: *biome*, *butterfly families*, *sex*, and *wingspan*; random variable: *site*)

| **Source** | **AIC** | **BIC** | **Loglik** | **Deviance** | **Chi-squared (χ²)** | **Df** | **P** |
| --- | --- | --- | --- | --- | --- | --- | --- |
| GLMM1 | 691.84 | 730.80 | -334.92 | 669.84 | 22.015 | 1 | **< 0.001** *** |
| GLMM3 | 711.86 | 747.27 | -345.93 | 691.86 | - | - | - |

**Table S8** Comparison of GLMM1 (includes response variables: *biomes*, *butterfly family*, *sex*, and *wingspan*; random variables: *sites*, and *butterfly species*) and GLMM4 (includes response variables: *biome*, *sex*, and *wingspan*; random variable: *site*, *butterfly species*)

| Source | AIC | BIC | Loglik | Deviance | Chi-squared (χ²) | Df |  | P |
| --- | --- | --- | --- | --- | --- | --- | --- | --- |
| GLMM1 | 691.84 | 730.80 | -334.92 | 669.84 | 17.949 | 4 |  | **0.001 **** |
| GLMM4 | 701.79 | 726.58 | -343.90 | 687.79 | - | - |  | - |

**Table S9** Comparison of GLMM1 (includes response variables: *biomes*, *butterfly families*, *sex*, and *wingspan*; random variables: *sites*, and *butterfly species*) and GLMM5 (includes response variables: *biome*, *butterfly family*, *sex*, *wingspan*, and *butterfly family***se*x; random variables: *site* and *butterfly species*)

| Source | AIC | BIC | Loglik | Deviance | Chi-squared (χ²) | Df |  | P |
| --- | --- | --- | --- | --- | --- | --- | --- | --- |
| GLMM1 | 691.84 | 730.80 | -334.92 | 669.84 | - | - |  | **-** |
| GLMM5 | 691.93 | 745.05 | -330.97 | 661.93 | 7.912 | 4 |  | 0.095 |

**Table S10** Comparison of GLMM1 (includes response variables: *biomes*, *butterfly families*, *sex*, and *wingspan*; random variables: *sites*, and *butterfly species*) and GLMM6 (includes response variables: *biome*, *butterfly family*, *sex*, *wingspan*, and *butterfly family***biome*; random variables: *site* and *butterfly species*)

| Source | AIC | BIC | Loglik | Deviance | Chi-squared (χ²) | Df |  | P |
| --- | --- | --- | --- | --- | --- | --- | --- | --- |
| GLMM1 | 691.84 | 730.80 | -334.92 | 669.84 | - | - |  | **-** |
| GLMM6 | 675.90 | 743.19 | -318.95 | 637.90 | 31.94 | 8 |  | **< 0.001 ***** |

**Table S11** Composition and number of butterflies collected across Australian biomes in 2023

| Species | Sex | Biome | | | Total Abundance |
| --- | --- | --- | --- | --- | --- |
|  |  | **Tropics** | **Sub-tropics** | **Temperate** |  |
| *Acraea terpsicore* | Female | 1 | 0 | 0 | 1 |
|  | Male | 2 | 0 | 0 | 2 |
| *Arrhenes dschilus* | Female | 11 | 0 | 0 | 11 |
|  | Male | 3 | 0 | 0 | 3 |
| *Belenois java* | Female | 0 | 1 | 0 | 1 |
|  | Male | 0 | 2 | 0 | 2 |
| *Candalides absimilis* | Female | 0 | 0 | 1 | 1 |
|  | Male | 0 | 0 | 0 | 0 |
| *Candalides cyprotus* | Female | 0 | 0 | 0 | 0 |
|  | Male | 0 | 0 | 1 | 1 |
| *Candalides erinus* | Female | 0 | 0 | 0 | 0 |
|  | Male | 0 | 1 | 0 | 1 |
| *Candalides hyacinthinus* | Female | 0 | 0 | 2 | 2 |
|  | Male | 0 | 0 | 14 | 14 |
| *Candalides margarita* | Female | 1 | 0 | 0 | 1 |
|  | Male | 0 | 0 | 0 | 0 |
| *Catopsilia pomona* | Female | 0 | 2 | 0 | 2 |
|  | Male | 1 | 4 | 0 | 5 |
| *Catopyrops florinda* | Female | 0 | 2 | 0 | 2 |
|  | Male | 0 | 2 | 0 | 2 |
| *Cephrenes augiades* | Female | 5 | 0 | 0 | 5 |
|  | Male | 0 | 0 | 0 | 0 |
| *Cepora perimale* | Female | 0 | 3 | 0 | 3 |
|  | Male | 1 | 3 | 0 | 4 |
| *Cethosia cydippe* | Female | 1 | 0 | 0 | 1 |
|  | Male | 1 | 0 | 0 | 1 |
| *Cethosia penthesilea* | Female | 0 | 0 | 0 | 0 |
|  | Male | 1 | 0 | 0 | 1 |
| *Cressida cressida* | Female | 0 | 1 | 0 | 1 |
|  | Male | 1 | 1 | 0 | 2 |
| *Cupha prosope* | Female | 6 | 1 | 0 | 7 |
|  | Male | 15 | 1 | 0 | 16 |
| *Danaus affinis* | Female | 0 | 0 | 0 | 0 |
|  | Male | 3 | 0 | 0 | 3 |
| *Danaus petilia* | Female | 0 | 1 | 0 | 1 |
|  | Male | 0 | 3 | 0 | 3 |
| *Danaus plexippus* | Female | 0 | 1 | 0 | 1 |
|  | Male | 0 | 21 | 2 | 23 |
| *Delias aganippe* | Female | 0 | 0 | 0 | 0 |
|  | Male | 0 | 1 | 0 | 1 |
| *Delias argenthona* | Female | 0 | 0 | 0 | 0 |
|  | Male | 0 | 1 | 0 | 1 |
| *Delias mysis* | Female | 3 | 0 | 0 | 3 |
|  | Male | 9 | 0 | 0 | 9 |
| *Deudorix diovis* | Female | 0 | 1 | 0 | 1 |
|  | Male | 0 | 1 | 0 | 1 |
| *Elodina angulipennis* | Female | 0 | 0 | 0 | 0 |
|  | Male | 0 | 3 | 0 | 3 |
| *Elodina parthia* | Female | 0 | 1 | 0 | 1 |
|  | Male | 0 | 0 | 0 | 0 |
| *Erysichton lineatus* | Female | 0 | 1 | 0 | 1 |
|  | Male | 0 | 0 | 0 | 0 |
| *Euploea corrinna* | Female | 1 | 2 | 0 | 3 |
|  | Male | 2 | 15 | 0 | 17 |
| *Euploea tulliolus* | Female | 3 | 0 | 0 | 3 |
|  | Male | 0 | 0 | 0 | 0 |
| *Eurema brigitta* | Female | 0 | 1 | 0 | 1 |
|  | Male | 0 | 0 | 0 | 0 |
| *Eurema hecabe* | Female | 4 | 0 | 0 | 4 |
|  | Male | 34 | 1 | 0 | 35 |
| *Eurema laeta* | Female | 2 | 0 | 0 | 2 |
|  | Male | 2 | 0 | 0 | 2 |
| *Eurema smilax* | Female | 0 | 0 | 1 | 1 |
|  | Male | 0 | 0 | 0 | 0 |
| *Graphium agamemnon* | Female | 0 | 0 | 0 | 0 |
|  | Male | 1 | 0 | 0 | 1 |
| *Graphium choredon* | Female | 0 | 0 | 0 | 0 |
|  | Male | 0 | 4 | 0 | 4 |
| *Hesperilla crypsigramma* | Female | 0 | 0 | 0 | 0 |
|  | Male | 0 | 2 | 0 | 2 |
| *Heteronympha merope* | Female | 0 | 0 | 0 | 0 |
|  | Male | 0 | 0 | 5 | 5 |
| *Hypochrysops pythias* | Female | 1 | 0 | 0 | 1 |
|  | Male | 0 | 0 | 0 | 0 |
| *Hypocysta irius* | Female | 0 | 0 | 0 | 0 |
|  | Male | 1 | 0 | 0 | 1 |
| *Hypocysta metirius* | Female | 0 | 0 | 0 | 0 |
|  | Male | 0 | 3 | 2 | 5 |
| *Hypolimnas alimena* | Female | 0 | 0 | 0 | 0 |
|  | Male | 1 | 0 | 0 | 1 |
| *Hypolimnas bolina* | Female | 1 | 0 | 0 | 1 |
|  | Male | 1 | 0 | 0 | 1 |
| *Hypolycaena phorbas* | Female | 1 | 0 | 0 | 1 |
|  | Male | 0 | 0 | 0 | 0 |
| *Jamides phaseli* | Female | 0 | 0 | 1 | 1 |
|  | Male | 0 | 0 | 1 | 1 |
| *Junonia hedonia* | Female | 0 | 0 | 0 | 0 |
|  | Male | 63 | 0 | 0 | 63 |
| *Junonia orithya* | Female | 1 | 0 | 0 | 1 |
|  | Male | 0 | 0 | 0 | 0 |
| *Junonia villida* | Female | 0 | 0 | 6 | 6 |
|  | Male | 0 | 0 | 4 | 4 |
| *Lampides boeticus* | Female | 0 | 2 | 1 | 3 |
|  | Male | 0 | 0 | 0 | 0 |
| *Leptotes plinius* | Female | 0 | 21 | 0 | 21 |
|  | Male | 0 | 57 | 0 | 57 |
| *Megisba strongyle* | Female | 0 | 0 | 0 | 0 |
|  | Male | 1 | 0 | 0 | 1 |
| *Melanitis leda* | Female | 11 | 12 | 0 | 23 |
|  | Male | 1 | 0 | 0 | 1 |
| *Mesodina halyzia* | Female | 0 | 0 | 0 | 0 |
|  | Male | 0 | 0 | 3 | 3 |
| *Mycalesis perseus* | Female | 42 | 0 | 0 | 42 |
|  | Male | 33 | 0 | 0 | 33 |
| *Mycalesis sirius* | Female | 25 | 0 | 0 | 25 |
|  | Male | 6 | 0 | 0 | 6 |
| *Mycalesis terminus* | Female | 66 | 0 | 0 | 66 |
|  | Male | 126 | 0 | 0 | 126 |
| *Mynes geoffroyi* | Female | 1 | 0 | 0 | 1 |
|  | Male | 0 | 0 | 0 | 0 |
| *Nacaduba berenice* | Female | 0 | 3 | 0 | 3 |
|  | Male | 0 | 8 | 0 | 8 |
| *Nacaduba cyanea* | Female | 1 | 0 | 0 | 1 |
|  | Male | 5 | 0 | 0 | 5 |
| *Nacaduba kurava* | Female | 0 | 0 | 0 | 0 |
|  | Male | 2 | 0 | 0 | 2 |
| *Neolucia mathewi* | Female | 0 | 0 | 3 | 3 |
|  | Male | 0 | 0 | 0 | 0 |
| *Neptis praslini* | Female | 2 | 0 | 0 | 2 |
|  | Male | 1 | 0 | 0 | 1 |
| *Netrocoryne repanda* | Female | 0 | 0 | 0 | 0 |
|  | Male | 1 | 0 | 0 | 1 |
| *Notocrypta waigensis* | Female | 1 | 0 | 0 | 1 |
|  | Male | 2 | 0 | 0 | 2 |
| *Ocybadistes flavovittatus* | Female | 0 | 0 | 1 | 1 |
|  | Male | 0 | 0 | 0 | 0 |
| *Ocybadistes hypomeloma* | Female | 0 | 0 | 0 | 0 |
|  | Male | 0 | 0 | 1 | 1 |
| *Ocybadistes knightorum* | Female | 0 | 0 | 1 | 1 |
|  | Male | 0 | 0 | 0 | 0 |
| *Ocybadistes walkeri* | Female | 0 | 1 | 6 | 7 |
|  | Male | 0 | 0 | 5 | 5 |
| *Pachliopta polydorus* | Female | 2 | 0 | 0 | 2 |
|  | Male | 1 | 0 | 0 | 1 |
| *Pantoporia consimilis* | Female | 5 | 0 | 0 | 5 |
|  | Male | 0 | 0 | 0 | 0 |
| *Papilio aegeus* | Female | 0 | 1 | 0 | 1 |
|  | Male | 1 | 4 | 1 | 6 |
| *Papilio ambrax* | Female | 0 | 0 | 0 | 0 |
|  | Male | 3 | 0 | 0 | 3 |
| *Papilio demoleus* | Female | 0 | 25 | 0 | 25 |
|  | Male | 0 | 3 | 0 | 3 |
| *Pelopidas agna* | Female | 2 | 0 | 0 | 2 |
|  | Male | 15 | 0 | 0 | 15 |
| *Pelopidas lyelli* | Female | 2 | 0 | 0 | 2 |
|  | Male | 5 | 0 | 0 | 5 |
| *Pieris rapae* | Female | 0 | 12 | 98 | 110 |
|  | Male | 0 | 38 | 266 | 304 |
| *Prosotas felderi* | Female | 0 | 1 | 0 | 1 |
|  | Male | 0 | 0 | 0 | 0 |
| *Psychonotis caelius* | Female | 0 | 0 | 0 | 0 |
|  | Male | 3 | 1 | 0 | 4 |
| *Sabera caesina* | Female | 0 | 0 | 0 | 0 |
|  | Male | 4 | 0 | 0 | 4 |
| *Sabera dobboe* | Female | 7 | 0 | 0 | 7 |
|  | Male | 2 | 0 | 0 | 2 |
| *Suniana sunias* | Female | 27 | 18 | 1 | 46 |
|  | Male | 20 | 5 | 1 | 26 |
| *Tagiades japetus* | Female | 0 | 0 | 0 | 0 |
|  | Male | 2 | 0 | 0 | 2 |
| *Taractrocera dolon* | Female | 0 | 0 | 1 | 1 |
|  | Male | 0 | 0 | 0 | 0 |
| *Telicota ancilla* | Female | 2 | 0 | 0 | 2 |
|  | Male | 11 | 0 | 0 | 11 |
| *Telicota mesoptis* | Female | 20 | 0 | 0 | 20 |
|  | Male | 43 | 0 | 0 | 43 |
| *Telicota ohara* | Female | 1 | 0 | 0 | 1 |
|  | Male | 1 | 0 | 0 | 1 |
| *Theclinesthes onycha* | Female | 1 | 31 | 0 | 32 |
|  | Male | 0 | 8 | 0 | 8 |
| *Theclinesthes sulpitius* | Female | 0 | 0 | 4 | 4 |
|  | Male | 1 | 0 | 0 | 1 |
| *Tisiphone abeona* | Female | 0 | 0 | 0 | 0 |
|  | Male | 0 | 0 | 6 | 6 |
| *Toxidia peron* | Female | 0 | 4 | 2 | 6 |
|  | Male | 0 | 4 | 7 | 11 |
| *Trapezites praxedes* | Female | 0 | 0 | 0 | 0 |
|  | Male | 0 | 0 | 1 | 1 |
| *Trapezites symmomus* | Female | 0 | 1 | 0 | 1 |
|  | Male | 0 | 0 | 0 | 0 |
| *Vanessa kershawi* | Female | 0 | 0 | 0 | 0 |
|  | Male | 0 | 0 | 1 | 1 |
| *Yoma sabina* | Female | 0 | 0 | 0 | 0 |
|  | Male | 1 | 0 | 0 | 1 |
| *Ypthima arctous* | Female | 11 | 0 | 0 | 11 |
|  | Male | 6 | 0 | 0 | 6 |
| *Zizina otis* | Female | 24 | 58 | 123 | 205 |
|  | Male | 58 | 70 | 167 | 295 |
| *Zizula hylax* | Female | 18 | 97 | 0 | 115 |
|  | Male | 30 | 158 | 0 | 188 |

**Table S12** Estimated probabilities of avian attacks by biomes, butterfly families, and sex

| **Category** | **Estimated probability** | **SE** | **95% confidence interval** |
| --- | --- | --- | --- |
| Sub-tropics | 0.675 | 0.051 | 0.569 - 0.766 |
| Temperate | 0.134 | 0.029 | 0.087 - 0.202 |
| Tropics | 0.727 | 0.047 | 0.625 - 0.809 |
| Hesperiidae | 0.284 | 0.039 | 0.213 - 0.366 |
| Lycaenidae | 0.499 | 0.037 | 0.427 - 0.571 |
| Nymphalidae | 0.673 | 0.040 | 0.590 - 0.747 |
| Papilionidae | 0.477 | 0.089 | 0.311 - 0.648 |
| Pieridae | 0.510 | 0.047 | 0.419 - 0.601 |
| Female | 0.545 | 0.039 | 0.468 - 0.620 |
| Male | 0.429 | 0.037 | 0.359 - 0.503 |
